# Correction to *Lancet Healthy Longev* 2021; 2: e352–61

**DOI:** 10.1016/S2666-7568(21)00185-9

**Published:** 2021-08

*Livingstone S, Morales DR, Donnan PT, et al. Effect of competing mortality risks on predictive performance of the QRISK3 cardiovascular risk prediction tool in older people and those with comorbidity: external validation population cohort study.* Lancet Healthy Longev *2021; **2:** e352–61*—This Article should have been published under a CC BY Open Access license. This correction has been made as of Aug 4, 2021.

